# Local activation of α2 adrenergic receptors is required for vagus nerve stimulation induced motor cortical plasticity

**DOI:** 10.1038/s41598-021-00976-2

**Published:** 2021-11-04

**Authors:** Ching-Tzu Tseng, Solomon J. Gaulding, Canice Lei E. Dancel, Catherine A. Thorn

**Affiliations:** School of Behavioral and Brain Sciences, University of Texas at Dallas, 800 W. Campbell Rd, Richardson, TX 75080 USA

**Keywords:** Motor cortex, Cortex

## Abstract

Vagus nerve stimulation (VNS) paired with rehabilitation training is emerging as a potential treatment for improving recovery of motor function following stroke. In rats, VNS paired with skilled forelimb training results in significant reorganization of the somatotopic cortical motor map; however, the mechanisms underlying this form of VNS-dependent plasticity remain unclear. Recent studies have shown that VNS-driven cortical plasticity is dependent on noradrenergic innervation of the neocortex. In the central nervous system, noradrenergic α2 receptors (α2-ARs) are widely expressed in the motor cortex and have been critically implicated in synaptic communication and plasticity. In current study, we examined whether activation of cortical α2-ARs is necessary for VNS-driven motor cortical reorganization to occur. Consistent with previous studies, we found that VNS paired with motor training enlarges the map representation of task-relevant musculature in the motor cortex. Infusion of α2-AR antagonists into M1 blocked VNS-driven motor map reorganization from occurring. Our results suggest that local α2-AR activation is required for VNS-induced cortical reorganization to occur, providing insight into the mechanisms that may underlie the neuroplastic effects of VNS therapy.

## Introduction

Enhancement of neuroplasticity within the motor system has been targeted as a potential therapy to restore the dysfunction that often arises following stroke and other neural injuries. Recent preclinical studies have demonstrated that vagus nerve stimulation (VNS) paired with specific rehabilitation training facilitates the recovery of motor function in rat models of stroke, traumatic brain injury, spinal cord injury, and peripheral nerve damage^1–6^. Early stage clinical studies have similarly suggested that VNS paired with rehabilitation exercises may improve upper limb function in stroke patients^7,8^. In both injured and in healthy rats, VNS paired with motor training consistently induces an expansion of the somatotopic map representation of task-relevant musculature within the motor cortex^4,9–12^. Recent findings further suggest that such motor cortical reorganization may be necessary for VNS-mediated enhancement of functional recovery to occur after injury^4^.

Though the mechanisms underlying VNS efficacy remain poorly understood, it has been shown that VNS-driven cortical reorganization depends on the coordinated activity of multiple neuromodulatory systems^4,10,11^. Neurotoxic lesions to cholinergic, serotonergic, or noradrenergic neuromodulatory systems block VNS-induced cortical map reorganization^10,11^, consistent with the critical role for each of these neuromodulators in supporting neocortical plasticity^13–17^. Noradrenaline (NA), in particular, has long been implicated in neuroplasticity and neuroprotection^13,15,18^, and noradrenergic signaling is known to play a critical role in VNS efficacy^11,19–24^. VNS increases the firing rates of noradrenergic neurons in the locus coeruleus (LC)^25^ and increases NA concentrations in the neocortex and hippocampus^20,26^. Moreover, lesions to the LC abolish the anti-depressant and seizure-attenuating effects of VNS^22,27^. These previous studies demonstrate that the beneficial effects of VNS depend on activation of the LC and intact noradrenergic signaling. However, the local mechanisms by which NA contributes to VNS-mediated neocortical plasticity remain unknown. Understanding the noradrenergic mechanisms of VNS is key to understanding its therapeutic limitations for neural injury recovery, as several common comorbidities can impair NA signaling, including Alzheimer’s disease, Parkinson’s disease, depression, and ADHD^28–31^.

Noradrenaline modulates synaptic transmission and plasticity throughout the CNS by acting at G-protein coupled α1, α2, and β adrenergic receptors (ARs). In sensory and prefrontal neocortices, ubiquitously expressed α2-ARs are known to regulate synaptic transmission^32^ and neuronal excitability^33–35^, and activation of this receptor subtype enhances attentional processes^36–38^. Given the importance of α2-ARs in regulating neocortical circuit function, we hypothesized that local activation of these receptors within the primary motor cortex (M1) may play a critical role in mediating the plasticity-promoting effects of VNS. To test this hypothesis, we used a pharmacological approach to block local α2-ARs in M1 during a VNS-paired motor paradigm previously shown to induce significant motor map reorganization. Our results demonstrate that antagonism of α2-ARs within M1 blocks VNS-induced motor map reorganization, suggesting that activation of these receptors critically contributes to the plasticity-promoting effects of VNS.

## Results

### VNS paired with motor performance increases the cortical map representation of task-relevant musculature

**Figure 1:**
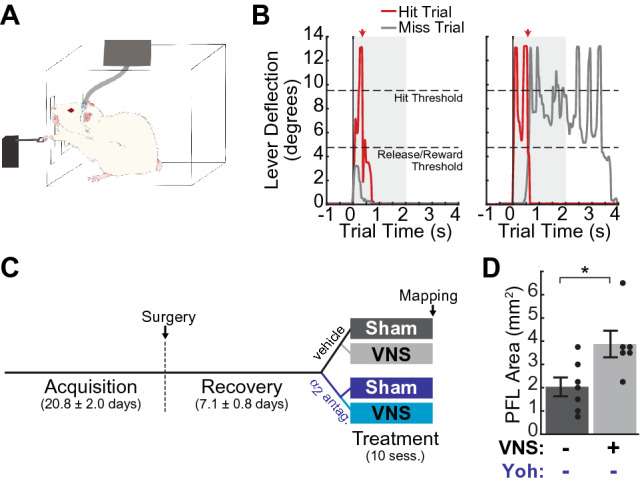
Experimental design. (**A**) Cartoon illustrating the skilled reaching lever-press task and VNS procedures. Rats were required to press and release a lever situated 2 cm outside the training booth within a 2 s window to receive reward (**B**) Example rewarded (Hit, red) trial and unrewarded (Miss, gray) trials from a single pretreatment session. Rats were required to press the lever past the “hit threshold” (9.5 degrees below horizontal) and return it to less than 4.75 degrees from horizontal (“Release/Reward Threshold”) in under 2 s to receive food reward. Unrewarded “Miss Trials” were those in which rats either failed to reach the hit threshold (gray trace, left) or failed to release the lever within the required time window (gray trace, right). (**C**) Experimental design and timeline. Female Sprague–Dawley rats were trained on the lever press task (Acquisition) prior to implantation of VNS cuffs and intracortical cannula. After surgery, rats were trained until stable performance was reestablished (Recovery), and then dynamically allocated to a treatment group. During Treatment, rats received 10 training sessions in which yohimbine (yoh) or control vehicle was infused into M1 30 min prior to behavioral training, and in which VNS or sham stimulation was paired with correct lever-press performance; sham|veh (n = 7), VNS|veh (n = 6), sham|yoh (n = 6), VNS|yoh (n = 6). (**D**) VNS paired with the lever pressing task significantly enlarged the task-relevant proximal forelimb (PFL) area in M1. *p < 0.05, Student’s unpaired t-test.

**Figure 2:**
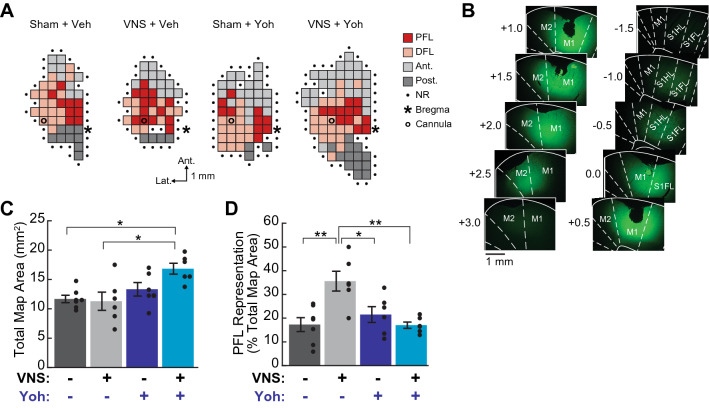
Cortical infusion of selective α2-AR antagonists blocked VNS-induced motor map reorganization. (**A**) Example cortical motor maps from subjects in each treatment group; representative maps are from subjects in each group that exhibited the median total map area and proximal forelimb (PFL) representation for that group. DFL: distal forelimb; Ant.: anterior body representation (vibrissa, head, or neck); Post.: posterior body representation (trunk, hindlimb, or tail); NR: non-responsive. (**B**) Cannula infusion of 1 μL sodium fluorescein dye was used to estimate drug diffusion within the cortex. (**C**) Total motor map area was significantly larger in VNS treated rats that received yohimbine (yoh) infusion compared to other groups. (**D**) To control for variation in total map size across subjects, subregion areas were normalized to total map area for each subject. VNS paired with lever training significantly enlarged the normalized PFL representation in vehicle-infused rats. In yohimbine-infused rats, VNS-driven PFL expansion was blocked. In (**C**), (**D**), filled circles indicate data for individual rats; error bars denote SEM; *p < 0.05, **p < 0.01, Tukey post hoc comparison.

Previous studies have shown that, in rats performing a highly skilled bradykinesia assessment task, VNS paired with successful lever pressing performance results in the expansion of the task-relevant proximal forelimb (PFL) map representation in the motor cortex contralateral to the trained limb^9–12,39^. In current study, rats were required to perform a simplified lever pressing task as previous described^40^ (Fig. 1A–C). We first verified that VNS-induced map reorganization was induced using this simplified training paradigm, by comparing the motor maps of sham- and VNS-treated rats that received intracortical vehicle infusions. We found that VNS paired with correct lever press performance significantly expanded the PFL representation within the cortical motor map (Fig. 1D; Comparison of PFL map area, sham|veh vs. VNS|veh: t(11) =  − 2.68; p = 0.022, Student’s unpaired t-tests). Moreover, the PFL representation was the only map region significantly impacted by VNS treatment (Supplementary Fig. 1; Comparison of map areas, sham|veh vs. VNS|veh: total map area: t(11) = 0.24, p = 0.811; distal forelimb (DFL): t(11) = 1.95, p = 0.077; anterior body (vibrissa + jaw + neck): t(11) = 0.383, p = 0.709; posterior body (trunk + hindlimb + tail): t(11) = 1.04, p = 0.323 Student’s unpaired t-tests). We note that in all animals, we were able to obtain complete cortical motor maps using ICMS (Fig. 2A; Supplementary Fig. 2), demonstrating that the cannula implantation and drug infusions did not significantly impact motor cortical somatotopy in our hands. Our results are highly consistent with previous studies indicating that VNS paired with a well-learned motor task selectively expands the cortical motor map representation of task-relevant musculature^9–12^, validating the training and treatment procedures used here.

### Intracortical infusion of α2-AR antagonists blocks VNS-induced motor map plasticity

To test whether α2-AR activation is necessary for VNS-induced motor cortical map reorganization, we infused the selective α2-AR antagonist yohimbine into M1 30 min prior to each training-paired VNS, or sham stimulation, session. Sodium fluorescein diffusion experiments suggest that our α2-AR antagonist infusions were likely to impact a large cortical area centered on M1, and to cover the majority of the forelimb map area in most animals (Fig. 2B). Two-way ANOVA was used to analyze the effects of VNS and yohimbine treatment on motor map plasticity. These analyses revealed a significant main effect of yohimbine treatment on total map area, as well as a main effect of yohimbine and a VNS x yohimbine interaction on distal and posterior subregions areas (Table 1). Post-hoc comparisons confirmed that the rats receiving both VNS and yohimbine treatments exhibited significantly larger total map areas compared to vehicle-treated groups (Fig. 2C; Supplementary Table 1), and that this effect was largely attributable to larger posterior body representations among the VNS|yoh group (Supplementary Table 1).

**Table 1:** Comparison of motor map areas across treatment groups.

	Vehicle		α2-AR antag.		2-way ANOVA		
	Sham	VNS	Sham	VNS	p_Drug_ [F_Drug_]	p_VNS_ [F_VNS_]	p_interaction_ [F_interaction_]
	Group mean (SEM)						
Total map area (mm^2^)	11.68 (0.63)	11.29 (1.55)	13.33 (1.15)	16.83 (0.93)	**0.003 [10.98]**	0.166 [2.05]	0.088 [3.20]
PFL area (mm^2^)	2.04 (0.41)	3.88 (0.57)	3.00 (0.66)	2.88 (0.29)	0.972 [0.00]	0.099 [2.98]	0.061 [3.91]
DFL area (mm^2^)	4.57 (0.51)	3.29 (0.38)	5.04 (0.51)	5.96 (0.27)	**0.002 [12.73]**	0.684 [0.17]	**0.021 [6.24]**
Anterior body area (mm^2^)	3.61 (0.50)	3.17 (1.11)	3.62 (0.49)	4.63 (0.49)	0.294 [1.16]	0.687 [0.17]	0.305 [1.14]
Posterior body area (mm^2^)	1.46 (0.31)	0.96 (0.39)	1.67 (0.50)	3.38 (0.54)	**0.007 [9.04]**	0.182 [1.91]	**0.019 [6.46]**
PFL representation (% total map)	17.28 (2.94)	35.59 (4.16)	21.50 (3.32)	17.03 (1.34)	**0.032 [5.3]**	**0.037 [4.93]**	**0.001 [13.38]**
DFL representation (% total map)	39.67 (4.21)	30.23 (2.70)	38.21 (2.77)	35.81 (2.15)	0.526 [0.42]	0.078 [3.43]	0.283 [1.22]
Anterior representation (% total map)	30.52 (3.28)	26.62 (5.53)	28.85 (4.77)	27.64 (2.77)	0.938 [0.01]	0.547 [0.37]	0.750 [0.10]
Posterior representation (% total map)	12.52 (2.57)	7.56 (3.22)	11.44 (2.88)	19.52 (2.28)	0.062 [3.90]	0.577 [0.32]	**0.028 [5.6]**

Within 24 h of the last training-paired treatment session, cortical motor maps were obtained using ICMS. ICMS-evoked movements at threshold amplitudes were classified as proximal forelimb (PFL), distal forelimb (DFL), anterior body (vibrissa, jaw, or neck), or posterior body (trunk, hindlimb, or tail) movements. VNS treatment resulted in an expansion of the task-relevant proximal forelimb representation, which was blocked by M1 infusion of α2-AR antagonists. Bold denotes statistical significance (p < 0.05).

Two-way ANOVA further revealed a trend toward a significant main effect of VNS on PFL area, as well as a trend toward a VNS x yohimbine interaction effect (Table 1). To examine subregion-specific effects and account for observed differences across treatment groups in total map size, we normalized PFL, DFL, anterior and posterior map areas by total map area for each subject. After normalization, two-way ANOVA revealed significant main effects of both VNS and yohimbine treatments, as well as a significant VNS x yohimbine interaction, that was specific to the task-relevant PFL motor map representation (Fig. 2D; Table 1; Supplementary Fig. 3). Post-hoc analyses revealed that while VNS treatment significantly increased the percentage of the motor map representing the PFL, infusion of yohimbine prior to VNS treatment blocked this effect (Comparison of PFL representations: sham|veh vs. VNS|veh: p = 0.002; VNS|veh vs. VNS|yoh, p = 0.002; sham|veh vs. VNS|yoh, p = 1.0; sham|yoh vs. VNS|yoh, p = 0.754; Tukey post hoc tests; see Supplementary Table 2 for full subregion-specific post-hoc results).

**Figure 3:**
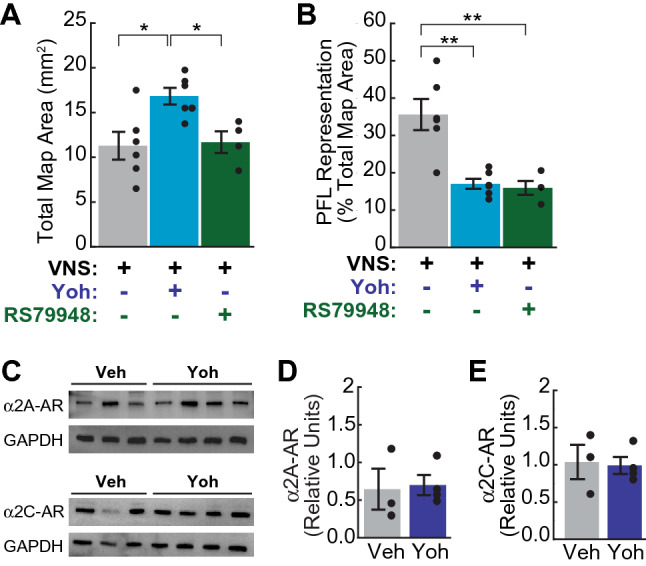
Cortical α2-AR antagonism blocks VNS-driven map reorganization without altering α2-AR protein expression. (**A**) Unlike yohimbine, the selective α2-AR antagonist RS79948 did not increase total motor map area in VNS-treated subjects (n = 4). (**B**) RS79948 infusion blocked VNS-induced expansion of the PFL representation. In (**A**), (**B**), *p < 0.05, **p < 0.01, Tukey post hoc comparison. (**C**–**E**) Intracortical infusion of yohimbine did not significantly alter the protein expression of α2A adrenoreceptor (α2A-AR, D) or α2C adrenoreceptor (α2C-AR, E) subtypes in M1. In (**C**), protein samples used to quantify expression of α2A-AR (top) and α2C-AR (bottom) were processed in parallel on separate gels. Control signals (GAPDH) were then detected from both membranes in parallel after stripping the primary antibodies of each α2-AR subtype. Images in (**C**) have been cropped to improve the clarity and conciseness of the presentation; full-length blots are presented in Supplementary Fig. 4. In D-E, α2A-AR (**D**) and α2C-AR (**E**) signal intensity is presented relative to same-column GAPDH intensity. Filled circles denote data for individual subjects; error bars denote SEM.

To control for potential off-target effects of yohimbine^41–43^, we tested a second, more selective α2-AR antagonist, RS79948^44–47^, in a smaller number of VNS treated rats (n = 4) that underwent treatment and ICMS mapping after the completion of the main study. Unlike yohimbine, RS79948 had no effect on total motor map area (Fig. 3A; Supplementary Table 3), suggesting that the overall map expansion we observed in the yoh|VNS treatment group may be unrelated to α2-AR antagonism. Yohimbine is known to additionally antagonize dopamine D2 receptors and agonize serotonin 5-HT1A receptors^43^, and both catecholamines have been linked to changes in motor map size^48,49^; these or other off-target effects may contribute to the enlarged maps observed in our yoh|VNS group. RS79948 was found to be as effective as yohimbine at blocking the VNS-induced expansion of the task-relevant PFL representation in M1 (Fig. 3B; Supplementary Table 3). Combined, these results suggest that antagonism of α2-ARs within M1 blocks VNS-driven motor map reorganization.

As xylazine exerts its anesthetic effects via agonism of α2-ARs, we also tested whether repeated intracortical administration of yohimbine resulted in neuronal changes that might have confounded our cortical mapping results. In sham-treated rats, yohimbine treatment did not result in any detectable functional effects on the ICMS motor map somatotopy (Fig. 2C,D; Table 1; Supplementary Tables 1–2). Nor did the microstimulation amplitude thresholds required to evoke movement during ICMS mapping differ across treatment groups (Supplementary Table 4). To directly test whether expression of α2-ARs was altered by repeated intracortical infusion of α2-AR antagonists, naïve rats received M1 infusions of either yohimbine (n = 4) or vehicle (n = 3) twice daily for 5 days. Western blot assays revealed that expression of α2A and α2C adrenoreceptor subtypes in M1 were not significantly different in vehicle and drug-treated rats (Fig. 3C–E; Supplementary Fig. 4; relative α2A-AR protein level: veh = 0.645 ± 0.27 vs. yoh = 0.699 ± 0.13, t(5) =  − 0.2, p = 0.853; relative α2C-AR protein level: veh = 1.037 ± 0.23 vs. yoh = 0.991 ± 0.11, t(5) = 0.19, p = 0.855; mean ± SEM, Student’s unpaired t-tests). These analyses indicate that α2-AR antagonism did not dramatically alter cortical α2-AR expression or motor map organization.

Taken together, our results suggest that activation of α2-ARs plays a critical role in VNS-induced cortical reorganization in the motor cortex.

### Neither VNS nor cortical α2-AR antagonism alters behavioral performance

**Figure 4:**
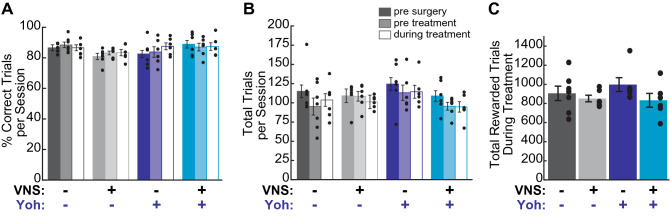
Differences in behavioral performance or VNS exposure cannot explain mapping results. (**A**) There was no difference across groups in average percent correct performance prior to surgery, before treatment, or during the ten treatment sessions, nor did treatment impact percent correct performance in any group. (**B**) No difference was observed across treatment groups in the average number of training trials performed per session during any epoch, nor did treatment significantly impact the number of trials performed within any treatment group. (**C**) The total number of rewarded trials did not differ across treatment groups during the Treatment period, indicating that VNS treated rats in both yohimbine- and vehicle-infused groups received equivalent exposure to VNS. Filled circles denote data for individual subjects; error bars denote SEM.

As differential behavioral performance could also potentially contribute to observed differences in VNS-driven cortical reorganization, we examined whether lever pressing performance varied across treatment groups. Prior to implantation of VNS electrodes and infusion cannula, we found no significant difference in learning rate across groups (Table 2). To test whether VNS or cortical α2-AR antagonism impacted behavioral performance, we compared the total trials performed and percent correct performance across training epochs and treatment groups. We found no difference across treatment groups in either performance measure during the training sessions prior to or during treatment (Fig. 4A,B; Table 2). Nor did we find a significant impact of training epoch on either total trials performed or percent correct performance for any treatment group (pre-treatment vs. treatment comparisons: percent correct performance: sham|veh: p = 0.075; VNS|veh, p = 0.941; sham|yoh: p = 0.210; VNS|yoh: p = 0.911; trials per session: sham|veh: p = 0.090; VNS|veh: p = 0.324 sham/drug: p = 0.795; VNS|yoh: p = 0.921; paired t-tests), confirming that the animals’ experience with the task did not differ prior to VNS or drug infusion, and that behavioral performance was not impacted by either treatment. Lever pressing speeds and trial durations did not differ across treatment groups during the pre-treatment and treatment periods (Table 2; Supplementary Fig. 5), further indicating that motor coordination during task performance was unaltered by local α2-AR antagonism or by VNS treatment. We also confirmed that all groups received an approximately equal number of VNS or sham stimulations during the 10-session treatment period (Fig. 4C; Table 2). These results demonstrate that differences in PFL map representations cannot be explained by differences in behavioral performance or VNS exposure across treatment groups. Combined, our findings show that VNS paired with motor training induces a significant reorganization of the somatotopic cortical motor map, and that this VNS-induced plasticity is blocked by the local administration of α2-AR antagonists.

**Table 2:** Lever press behavior did not differ across treatment groups and was not impacted by VNS or α2-AR antagonist infusions.

	Vehicle		α2-AR antag.		Two-way ANOVA		
	Sham	VNS	Sham	VNS	p_Drug_ [F_Drug_]	p_VNS_ [F_VNS_]	p_interaction_ [F_interaction_]
	Group mean (SEM)						
Sessions to criteria	16.3 (2.1)	21.2 (5.6)	21.3 (10.7)	24.2 (6.7)	0.557 [0.36]	0.573 [0.33]	0.881 [0.02]
**Pretreatment performance**							
Trials per session	95.1 (11.0)	108.4 (6.2)	113.1 (10.0)	95.5 (5.0)	0.774 [0.08]	0.809 [0.06]	0.092 [3.12]
Percent correct performance	88.5 (1.8)	83.2 (1.2 )	83.8 (3.5)	87.1 (2.5)	0.867 [0.03]	0.665 [0.19]	0.088 [3.21]
Lever pressing speed (deg/s)	137.7 (2.2)	144.3 (6.2)	151.9 (7.7)	150.5 (5.3)	0.375 [0.82]	0.816 [0.06]	0.724 [0.13]
Trial duration (ms)	445.1 (24.6)	400.3 (21.3)	475.3 (36.3)	528.5 (45.5)	0.244 [1.43]	0.950 [0.0]	0.467 [0.55]
**Treatment performance**							
Trials per session	103.7 (8.2)	101.2 (3.4)	114.4 (8.4)	94.7 (6.8)	0.769 [0.088]	0.134 [2.427]	0.239 [1.467]
Percent correct performance	86.6 (1.9)	83.4 (2.1)	87.5 (2.3)	87.4 (2.5)	0.280 [1.228]	0.459 [0.570]	0.499 [0.473]
Lever pressing speed (deg/s)	133.9 (3.4)	142.9 (6.3)	149.9 (6.3)	160.3 (7.0)	0.168 [2.04]	0.413 [0.70]	0.954 [0.0]
Trial duration (msec)	421.9 (18.2)	421.3 (25.3)	473.3 (36.7)	464.8 (46.2)	0.478 [0.52]	0.946 [0.0]	0.952 [0.0]
Total stimulations delivered	905.7 (75.7)	851.7 (34.4)	997.3 (72.3)	832.8 (72.0)	0.593 [0.294]	0.118 [2.652]	0.420 [0.677]

## Discussion

Prior studies have shown that VNS phasically activates the LC, and that cortical noradrenergic depletion inhibits VNS-driven motor map reorganization^11,25^. Here, we replicate and extend this prior work, demonstrating that selective antagonism of α2-ARs within the motor cortex during VNS treatment is sufficient to block VNS-induced plasticity. Our results suggest that the activation of α2-ARs following VNS-driven noradrenaline release plays a critical role in the induction of motor cortical plasticity that may underlie VNS efficacy during motor rehabilitation.

Cortical map reorganization in healthy animals is generally found to be correlated with learning-related improvements in sensory discrimination or motor performance^50–54^. Once a task becomes well-learned, however, cortical maps revert to a macro structure that closely resembles that of naïve animals while behavioral proficiency is maintained^9,50,55^, suggesting that map expansion per se is not necessary to maintain good performance. Our finding that VNS-induced map reorganization is not accompanied by a change in behavioral performance is supported by several prior studies that utilize a similar training-paired VNS treatment protocol^9–12^, and is consistent with literature suggesting that after task mastery has been achieved, overrepresentation of task-relevant musculature is not associated with further performance improvement^56^.

Cortical motor map reorganization also accompanies successful motor rehabilitation following stroke and other neural injuries^4,57–59^. Importantly, disruption of cortical plasticity processes during recovery both disrupts map reorganization and impairs performance improvement^4,52,59^, suggesting that map plasticity reflects neuronal plasticity processes that are functionally relevant for successful rehabilitation. In animals recovering from peripheral nerve injury (PNI), VNS paired with rehabilitation training was recently shown to induce an expansion of the M1 representation of task-relevant musculature^4^ similar to that observed in healthy subjects. Cortical cholinergic depletion, known to block VNS-induced map reorganization in healthy rats^10^, was found to block both map reorganization and performance recovery in the PNI subjects^4^, providing additional evidence that VNS-driven map reorganization likely reflects functionally relevant plasticity processes.

In the current study, we find that α2-AR antagonism similarly inhibits VNS-induced map reorganization in healthy subjects. A key question arising from this result, then, is whether cortical α2-ARs critically contribute to VNS-enhanced motor rehabilitation following neural injury. Given the ubiquitous expression of α2-ARs within the neocortex, and the well-known involvement of these receptors in synaptic transmission and plasticity, future studies will be critical to determine the specific cellular and circuit mechanisms by which α2-ARs may contribute to VNS efficacy. For example, activation of α2-AR autoreceptors within the neocortex is known to suppress the presynaptic release of NA by LC terminals^32,60^. Antagonism of these autoreceptors might thus result in higher-than-normal NA release in response to VNS. The idea that excessive NA release could block VNS-driven cortical plasticity is consistent with prior studies that report an inverted U-shaped curve for VNS efficacy^12,61^. In these studies, moderate stimulation amplitudes effectively induce cortical reorganization, but high intensities fail to induce plasticity. Whether NA is specifically involved in this phenomenon has not yet been determined, but one possibility is that excessive activation of low-affinity β adrenergic receptors (β-ARs) leads to the desensitization or internalization of this receptor subtype. β-AR activation is known to promote LTP at glutamatergic cortical synapses^62–64^, thus, a loss of signaling at these receptors could diminish the ability of VNS to drive neocortical plasticity.

Antagonism of heterosynaptic α2-ARs may also contribute to our findings. In sensory and motor cortices, activation of heterosynaptic α2-ARs has been shown to promote dendritic excitability, increasing synaptic integration and enhancing dendritic calcium activity that is thought to drive long-term plasticity^33,65,66^. In prefrontal cortex (PFC), α2-AR activation similarly enhances dendritic excitability, resulting in the persistent firing of pyramidal neurons that is thought to underlie working memory^36–38^. Activation of postsynaptic α2-ARs located on apical dendrites of cortical pyramidal neurons increases pyramidal neuron excitability via G_i_-coupled downregulation of cAMP leading to the closure of cyclic nucleotide-gated HCN channels^33–35,38,67,68^. The closure of HCN channels increases membrane resistance and the temporal summation of dendritic inputs, increasing the probability of dendritic Ca^++^ events^33,66^, persistent firing^38,66,69^ and synaptic plasticity^70^. In our study, α2-AR antagonism in M1 during task performance would be expected to reduce excitability, synaptic integration, and dendritic Ca^++^ signaling among HCN-expressing pyramidal cells, which could impair the ability of VNS to drive plasticity in the network. Further research is needed to clarify whether and how blockade of cortical α2-ARs impacts VNS-driven noradrenergic signaling and synaptic plasticity in the neocortex.

Inflammatory pathways may also play a role in the NA-dependent pro-plasticity effects of VNS. In the CNS, NA has been shown to promote β-AR dependent neuroprotective effects; these include inhibition of microglial activation, reduced production of proinflammatory factors, and activation of neurotrophic pathways^18,71–73^. Moreover, VNS has recently been shown to engage anti-inflammatory signaling pathways in animal models of neurodegenerative disease^74,75^. As α2-AR antagonism has been shown to increase local noradrenaline release, disrupted β-AR activation could contribute to disrupted neuroprotective signaling and decreased VNS efficacy. In our study, chronic implantation of intracortical cannula would certainly be expected to trigger strong inflammatory responses in M1. We note that all subjects in our study underwent cannulation, and that, in the absence of α2-AR antagonists, VNS was nonetheless effective at inducing cortical motor map plasticity. These results suggest that any inflammatory response present at the time of VNS delivery was not sufficient to interfere with VNS efficacy in our study. We cannot, however, rule out the possibility that drug-mediated disruptions in inflammatory signaling may have contributed to the blockade of VNS-induced plasticity that we observed following α2-AR antagonism. As inflammation is an important component of neural injury and recovery, future studies will be critical in determining the extent to which VNS-enhanced motor rehabilitation depends on modulation of neuroinflammatory pathways.

VNS has been reported to enhance a variety of cortical and subcortical memory processes, consistent with activation of multiple, broadly projecting, plasticity promoting neuromodulatory systems^76^. In the neocortex, VNS paired with sensory cues or specific movements drives task-dependent map plasticity^40,77–80^, while training alone or unpaired VNS fail to induce cortical reorganization^4,9,77,81^. VNS delivery during item presentation has been associated with increased memory retention and improved working memory performance^82–85^. These effects on memory and cognition are correlated with VNS-driven hippocampal plasticity, including induction of LTP^86,87^ and increased progenitor cell proliferation in the dentate gyrus^88–90^. Recent studies have also demonstrated that VNS paired with conditioned cues enhances the extinction of conditioned fear^91,92^ and the extinction of cued drug-seeking^93^. This VNS-enhanced extinction memory is associated with changes in neuronal activation and synaptic plasticity that are specific to the prefrontal cortex and basolateral amygdala^93^. Combined, these studies at once highlight both the broad potential impacts of VNS and the region-specific plasticity obtained when VNS is temporally paired with specific cues or behaviors. Such findings have attracted significant interest in VNS-driven targeted neuroplasticity as a potential treatment for a variety of neurological disorders, including stroke, tinnitus, and PTSD^76^.

VNS paired with motor rehabilitation training is increasingly under investigation to improve therapeutic outcomes in patients recovering from neural injuries. While VNS efficacy is known to depend on the coordinated release of neuromodulators within the neocortex, the specific cellular and circuit mechanisms involved remain poorly understood. Our results demonstrate that activation of α2-ARs plays a critical role in the induction of motor cortical reorganization by VNS, which is thought to be key to the recovery of motor function following stroke and other neural injuries. These findings may have significant clinical implications for the use of VNS as a plasticity promoting therapy, as many commonly prescribed medications impact noradrenergic signaling, including monoamine reuptake inhibitors, β-blockers, α-blockers, and α2 agonists. Moreover, among older populations most at risk for stroke, noradrenergic depletion due to Alzheimer’s disease, Parkinson’s disease, or other dementias is not uncommon^28,29^. The current study underscores the importance of cortical noradrenergic signaling in VNS efficacy and opens broad possibilities for future research into the cellular mechanisms by which α2-AR activation impacts motor cortical network dynamics and plasticity.

## Methods

All procedures were approved by the University of Texas Institutional Animal Care and Use Committee in accordance with the National Institutes of Health guide for the care and use of laboratory animals. Experiments were designed, conducted, and reported in compliance with ARRIVE guidelines.

### Animal subjects and experimental design

A total of fifty-four adult female Sprague–Dawley rats, 10–12 weeks old (251.3 ± 3.8 g) at study start, were used in these experiments. Female rats were used because substantial previous studies have shown that VNS paired with the motor behavior used in this study induces cortical plasticity in this sex^9–12,94^. Rats were pair housed throughout the study in a 12:12 h reversed light cycle room (lights on at 6:00 pm) and all procedures were performed during the dark cycle. During behavioral training, rats were food restricted to approximately 90–95% of their free-feeding weight.

To test the effects of local α2-AR antagonism on VNS-induced cortical plasticity, rats were trained on a skilled reaching lever press task. After reaching proficiency on the task, VNS cuff electrodes were chronically implanted around the left cervical vagus nerve and intracortical infusion cannula were implanted targeting the forelimb region of M1. After surgical recovery, rats returned to training until stable behavioral performance was reestablished on the lever press task. Rats were then assigned to one of four treatment groups using dynamic allocation^95^. A two-factor study design was used to test the effects of training-paired VNS (or sham) treatment and intra-M1 infusion of the α2-AR antagonist, yohimbine (or vehicle).

To control for off-target effects of yohimbine, additional lever-trained subjects were included in a control group that received training-paired VNS treatment along with intracortical infusions of the more selective α2-AR antagonist, RS79948. Rats that received RS79948 were the last to begin behavioral training and were not randomly assigned to this treatment group. Rats in the RS79948 group were trained in parallel with rats included in all other treatment groups, however, VNS and RS79948 infusions, and ICMS motor cortical mapping procedures, occurred 4–10 weeks after the main study was completed. Experimenters conducting the behavioral training, VNS, and mapping procedures remained blinded to the treatment assignments of all rats throughout the study, including those of the RS79948 group.

In total, forty-five rats underwent behavioral training. Sixteen subjects were excluded from the study due to health complications prior to (n = 1) or during recovery from (n = 2) surgery, failure to meet behavioral performance criteria during task Acquisition (n = 2) or Recovery (n = 1), failure of implanted cannula during Treatment (n = 4), or failure of implanted cuff electrodes (n = 6). Twenty-nine rats successfully completed the study and are included in the behavior and motor mapping analyses: twenty-five in the main treatment groups (sham|veh: n = 7; VNS|veh: n = 6; sham|yoh: n = 6; VNS|yoh: n = 6), and an additional four in the VNS|RS79948 control group. Additional naïve subjects (n = 9) were included in further control experiments as described below.

### Behavioral training

Rats were trained on a simplified version of a previously published automated lever pressing task^9–12,39^, in which they were required to fully depress and release a lever positioned 2 cm outside a MotoTrak behavioral chamber (Vulintus Inc., Dallas, TX) to receive a food reward (Fig. 1A,B). The slot through which the rats accessed the lever was positioned next to a cage divider, which ensured that the rats performed this skilled-reaching lever-press task with their right forelimb. Lever position was continuously monitored at 100 Hz and ranged from 0 (no force applied) to 13 degrees below horizontal (maximum lever depression). Rats received a 45 mg food pellet (Bio-Serv, Flemington, NJ) upon successfully depressing (to greater than 9.5 degrees from horizontal) and then releasing (return to less than 4.75 degrees from horizontal) the lever within a 2 s window (Fig. 1B). Prior to the training, each rat was acclimated to handling during 3–5 daily 10-min acclimation sessions, then habituated to the training environment during two 30-min habituation sessions, delivered on consecutive days, in which they were allowed to freely explore the MotoTrak booth. After habituation, rats received two 30-min behavioral training sessions on the lever-press task per day, five days per week, with at least 1-h rest in the homecage between same-day sessions. Training on the task was performed in several stages. In stage 1, the lever was located inside the booth and rats were rewarded for making progressively larger lever depressions. Once the rats could perform full lever depression for 100 trials/day for two consecutive days, the rats moved on to stage 2, in which reward was received after releasing the lever following a full depression. The Acquisition training phase began with stage 3, in which the lever was progressively moved from the interior to the exterior of the training booth, until the final position of 2 cm outside the booth was reached. Daily behavioral training continued until performance criteria were reached (> 55 trials and > 65% correct per session, for 8/10 consecutive training sessions). Surgery was then performed to implant vagus nerve cuff electrodes and intracortical guide cannula. After 3–7 days of rest following surgery, animals received additional Recovery training sessions to acclimate to the stimulating cable and to re-establish criterial behavioral performance. Animals were then assigned to a treatment group using dynamic allocation^95^ and received 10 final treatment sessions in which intracortical drug or vehicle infusions and training-paired VNS or sham stimulation were administered. The experimental design and timeline are shown in Fig. 1C.

### Surgical procedures

Rats were anesthetized with a cocktail of ketamine hydrochloride (50 mg/kg), xylazine (20 mg/kg), and acepromazine (5 mg/kg) injected intraperitoneally and received supplementary doses as need to maintain stable anesthesia throughout the procedures. Implantation of vagus nerve cuff electrodes was performed as previously described^3,10,96^. Briefly, an incision was made in the neck and the left vagus nerve exposed by blunt dissection of the overlying muscles. The vagus nerve was isolated from the carotid artery with blunt dissection and placed inside a bipolar stimulating cuff electrode with low impedance (< 5 kΩ) platinum-iridium leads^97–99^. Lead wires were tunneled subcutaneously toward the skull and attached to a 2-channel connector. Proper cuff function was validated by stimulating the vagus nerve using a 10 s train of 0.8 mA, 100 µs biphasic pulses delivered at 30 Hz to elicit a brief cessation of breathing and drop in SpO_2_ consistent with the Hering-Breuer reflex^100^. The neck incision was then sutured, and the animal’s head stabilized in a stereotaxic frame. Three anchor screws were inserted into the skull at points near the lambdoid suture and the two-channel connector was cemented in place using acrylic. A 22-gauge steel guide cannula (Plastics1, Roanoke, VA) was targeted to the forelimb area of left primary motor cortex (AP: + 0.5 mm, ML: − 2.5 mm from bregma, DV: − 1.0 mm from pial surface) and cemented in place with acrylic. The scalp incision was sutured, a topical antibiotic cream was applied to both incision sites, and animals were provided with Rimadyl (carprofen, 2 mg/5 g tablet) and Baytril (enrofloxacin, 0.5 mg/5 g tablet) tablets (Bio-Serv, Flemington, NJ) for three days during recovery.

### VNS parameters

After recovery from surgery, rats continued behavioral training until criterial performance was re-established on the task. Prior to treatment group assigment, implanted cuff function was validated by delivering 1–5 VNS test stimulations to ensure that implanted cuff impedance was between 3 and 4 kΩ and that stimulation provoked no overt behavioral response. Rats in which electrode function could not be validated prior to group assignment were either dropped from the study (n = 2) or included in the veh|sham treatment group (n = 4). Rats with behavioral responses to stimulation were excluded from the study (n = 2). Subjects then received 10 additional training sessions in which VNS, or sham stimulation, was delivered during rewarded trials immediately following each correct lever press-and-release. VNS parameters were identical to those shown in previous studies to effectively induce motor cortical map reorganization^9–12^: VNS was delivered as a 500 ms train of 15 pulses (0.8 mA, 100 μsec biphasic pulse width) at 30 Hz. Sham treated rats were connected to the stimulator cables and received identical behavioral training as VNS groups, but no stimulation was delivered during the training sessions. During each training-paired VNS treatment session, cuff function was continuously monitored using a digital oscilloscope to ensure that implanted device impedance remained within 3–4 kΩ. VNS-treated rats were excluded from the study if cuff function was lost during the Treatment period (n = 1).

### Drug administration

Thirty minutes prior to each of the final 10 training-paired VNS or sham treatment sessions, an α2-AR antagonist, or drug-free vehicle, was infused into the motor cortex to assess the necessity of α2-AR activation in VNS-dependent cortical reorganization. For the main study, the α2-AR antagonist yohimbine (Acros Organics, Fair Lawn, NJ) was used (sham|veh, n = 7; VNS|veh, n = 6; sham|yoh, n = 6; VNS|yoh, n = 6). As yohimbine has been found to exhibit off-target effects at higher concentrations^41,43^, we also tested the highly selective α2-AR antagonist RS79948^44–47^ (Tocris Bioscience, Bristol, United Kingdom) in an additional group of VNS-treated rats (n = 4). Neurological effects of yohimbine and RS79948 following intracranial infusions are well supported by prior electrophysiological and behavioral studies^37,38,101–105^. Here, we utilized the same intracranial infusion parameters adopted in previously published rodent studies^102,104^. A total volume of 1 μL of yohimbine (5 μg/μL in 40% saline + 60% DMSO), RS79948 (2 μg/μL in 40% saline + 60% DMSO) or vehicle (40% saline + 60% DMSO) was infused through the implanted cannula at a rate of 0.25 μL/min. Cannula placements in M1 were stereotaxically confirmed during surgery, and fluid flow was validated during each infusion.

To estimate the probable spread of intracortically infused compounds within M1, a 1% solution of sodium fluorescein salt (Sigma Aldrich, St Louis, MO, USA) was intracortically infused at the M1 target coordinates in two untrained rats (Fig. 1D). Sodium fluorescein was prepared in the same 40% saline + 60% DMSO solvent and has similar chemical properties as the compounds used in this study, including similar molecular weight (sodium fluorescein MW: 376.27 g/mol; yohimbine MW: 354.4 g/mol; RS79948 MW: 401 g/mol), charge (0 for all drugs), and low polar surface area (sodium fluorescein: 78.8 Å^2^; yohimbine: 65.6 Å^2^; RS79948: 58.2 Å^2^). These structural similarities suggest that sodium fluorescein should provide an effective estimate of yohimbine and RS79948 diffusion within neural tissue, though we note that differences in steric properties across the three compounds may nonetheless contribute to differential cortical diffusion. Dye was infused using identical parameters as those used during daily drug administration (volume: 1 μL, rate: 0.25 μL/min) and allowed to diffuse for 30 min. Rats were then deeply anesthetized with 0.5 mL sodium pentobarbital and phenytoin solution (Somnosol, Henry Schein Animal Heath, Dublin OH) delivered intraperitoneally, and transcardially perfused with 120 mL ice-cold PBS followed by 120 mL 4% paraformaldehyde. Brains were removed and fixed with 4% paraformaldehyde for 24 h, followed by cryoprotection with 30% sucrose for 48–72 h. Coronal sections were prepared with a Leica cryostat at 40 μm thickness.

### Western blot

To test whether repeated intracortical infusions of α2-AR antagonists altered receptor expression levels in M1, 7 naïve female Sprague–Dawley rats were implanted with intracortical infusion cannula targeting the left forelimb area of M1, as described above. After recovery from surgery, rats were infused with yohimbine (n = 4) or drug-free vehicle (n = 3) twice a day for 5 consecutive days. Solutions and infusion parameters were identical to those used in the main treatment groups, described above. Twenty-four hours after the final infusion, animals were anesthetized and sacrificed by decapitation; brains were quickly removed and flash frozen on dry ice. For each subject, four 1 mm diameter tissue punches were collected from M1 and homogenized in lysis buffer (10 mM Tris, pH 7.5, 150 mM NaCl, 1 mM EDTA, and 1% NP-40) containing protease and phosphatase inhibitors (Roche, Basel, Switzerland). Protein concentration in each sample was quantified using a Pierce BCA Protein Assay kit (Thermo Scientific, Waltham, MA). A total of 25 μg of protein per sample was loaded into 10% SDS-PAGE gels and separated by electrophoresis. Proteins were then transferred onto 0.45 µm pore size PVDF membranes (MilliporeSigma, Burlington, MA) using a wet tank transfer system (Bio-Rad Laboratories, Hercules, CA) at 150 V for 1.5 h on ice. The membranes were then blocked with 5% non-fat dry milk in TBST Tris buffer (0.1 M Tris, 0.9% NaCl, 0.1% Tween-20) for 1 h at room temperature, washed with TBST three times and hybridized with primary antibody (Anti-α2A Adrenergic receptor, 1:200, Proteintech, Cat No: 14266-1-AP; Anti-α2C Adrenergic receptor, 1:500, Sigma, SKU: SAB4500566) overnight at 4 °C. The membranes were washed three times with TBST and hybridized with secondary antibodies (Anti-rabbit IgG, HRP-linked antibody, 1:10,000, Cell Signaling Technology) for 1 h at room temperature. Finally, the membranes were washed with TBST (3x) and signals were detected with Immobilon Western Chemiluminescent HRP substrate (MilliporeSigma, Burlington, MA) and visualized with a Bio-Rad ChemiDoc Touch. Membranes were then stripped using a mild stripping buffer (199.8 mM glycine, 3.5 mM sodium dodecyl sulfate, 0.01% Tween-20, pH = 2.2) and reprobed with control antibody (GAPDH Polyclonal antibody, 1:5000, Proteintech, Cat No: 10494-1-AP). Signal band intensity was analyzed using ImageJ; for each subject, α2A-AR and α2C-AR target band intensities were normalized to their respective same-gel GAPDH signal intensities.

### Intracortical microstimulation (ICMS) mapping

Within 24 h of the final treatment-paired training session, rats underwent ICMS mapping of the left motor cortex to derive functional somatotopic maps as previous described^9–12^. Rats were anesthetized with ketamine hydrochloride (70 mg/kg) and xylazine (5 mg/kg) injected intramuscularly and placed in a stereotaxic frame. Prior to mapping, cuff electrode function was validated in VNS-treated subjects by applying a 10 s train of 100 µs biphasic pulses (amplitude = 0.8 mA; pulse frequency = 30 Hz) to elicit the Hering-Breuer reflex. Rats in which no reflex was evoked were excluded from the study (n = 1). VNS headcaps and chronically implanted cannula were surgically removed. To prevent swelling, a small incision was made in the cisterna magna. A craniotomy and durotomy were performed to expose the left motor cortex (ca. + 4.0 to − 3.0 mm anterior/posterior and ca. + 0.2 to + 5 mm lateral to bregma) and a low impedance tungsten electrode (300–500 kΩ, FHC, Bowdoin, ME) was stereotaxically lowered 1.8 mm into motor cortex at multiple sites in a grid pattern with 0.5 mm spacing. Silicone fluid was used to cover the exposed cortical surface. Microstimulation consisted of a 40 ms train of ten 200 μsec monophasic cathodal pulses delivered at 300 Hz. Stimulation intensity was gradually increased from 0 to 200 μA until a movement was first observed or maximal amplitude was reached. Threshold-evoked movements were classified as proximal forelimb, distal forelimb, vibrissa, jaw, neck, trunk, hindlimb, or tail. If no movement was observed at 200 μA, responses were evaluated at 1.6 mm and 2.0 mm electrode depths to allow for variations in cortical thickness. Stimulation sites within the grid were tested in random order, and the borders of motor cortex were defined by unresponsive sites at 200 μA amplitude at all three depths. ICMS was accomplished with two experimenters. One experimenter determined the random placement of the stimulation electrode. The second experimenter, blinded to both the location of the electrode and to the treatment condition of the rat, delivered the stimulation and determined the primary movement and threshold stimulation amplitude.

### Statistical analysis

Statistical analyses were performed in Python using the Pandas library^107^, SciPy library^108^, and the statsmodels package^109^. All summary data are reported as mean ± standard error of the mean (SEM).

To confirm that the simplified behavioral training paradigm used in this study was able to replicate VNS-induced cortical plasticity as reported in the literature, cortical map areas from the two vehicle treated groups were first compared using Student’s unpaired t-test. For ICMS mapping analyses in the main study, ICMS mapping results were compared across all treatment groups using two-way analysis of variance (ANOVA), followed by Tukey post hoc tests. For all motor map comparisons, significant differences are reported if p < 0.05.

Behavioral performance during the pre-treatment and treatment periods was compared across groups using two-way ANOVA. Behavioral parameters examined included average trials per session, average percent correct performance per session, average lever pressing speed, average trial duration, and total VNS or sham stimulations delivered during treatment. For all behavioral comparisons, the treatment period consisted of the 10 sessions in which VNS or sham stimulation was paired with correct task performance, while the pre-treatment period consisted of the 6 training sessions immediately prior to treatment. Within each treatment group, changes in behavioral performance between the pre-treatment and treatment periods were assessed using paired two-sample t-tests. For all behavioral comparisons, significant differences are reported if p < 0.05.

## Supplementary Information

Supplementary Information.

## Data Availability

The datasets generated during and/or analysed during the current study are available from the corresponding author on reasonable request.

## Abbreviations

α2-ARs: Alpha-2 noradrenergic receptors
ICMS: Intracortical microstimulation
M1: Primary motor cortex
NA: Noradrenaline
PFL: Proximal forelimb
VNS: Vagus nerve stimulation
veh: Vehicle
yoh: Yohimbine
