# Supplementary material for: Local activation of α2 adrenergic receptors is required for vagus nerve stimulation induced motor cortical plasticity
Source: Sci Rep. 2021 Nov 4;11:21645. doi: 10.1038/s41598-021-00976-2 (PMC8568982; doi:10.1038/s41598-021-00976-2)
Supplement: Supplementary file 1 — Supplementary Information. [file 41598_2021_976_MOESM1_ESM.pdf]

SUPPLEMENTARY MATERIAL

Local activation of α2 adrenergic receptors is required for vagus nerve stimulation induced motor cortical plasticity

Ching-Tzu Tseng, Solomon J. Gaulding, Canice Lei E. Dancel, Catherine A. Thorn*

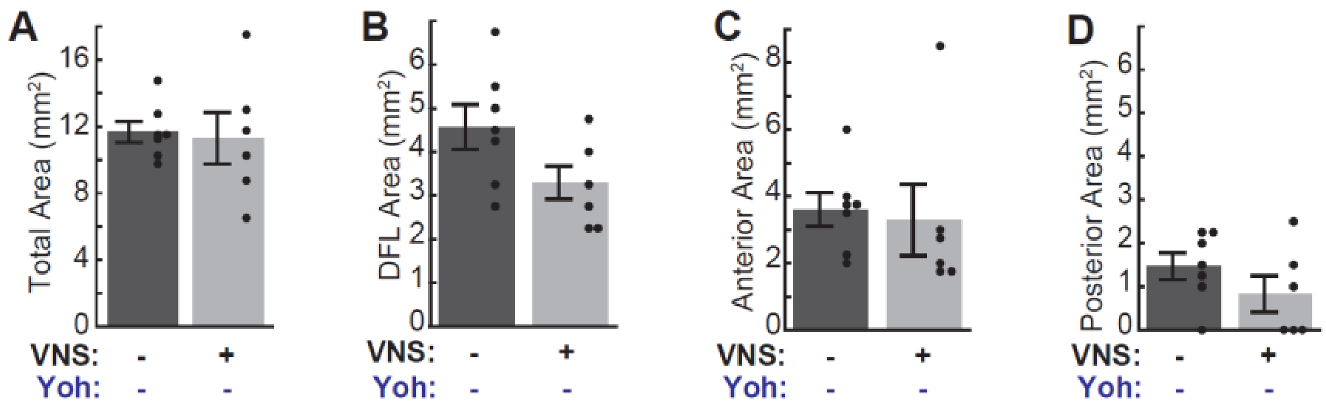

Supplementary Figure 1. Using the single-press behavioral training paradigm, press-paired VNS treatment did not induce significant changes in **(A)** total motor map area, nor in subregion areas associated with **(B)** distal forelimb (DFL), **(C)** Anterior body (vibrissa, neck, and jaw), **(D)** Posterior body (trunk, hindlimb, and tail) representations.

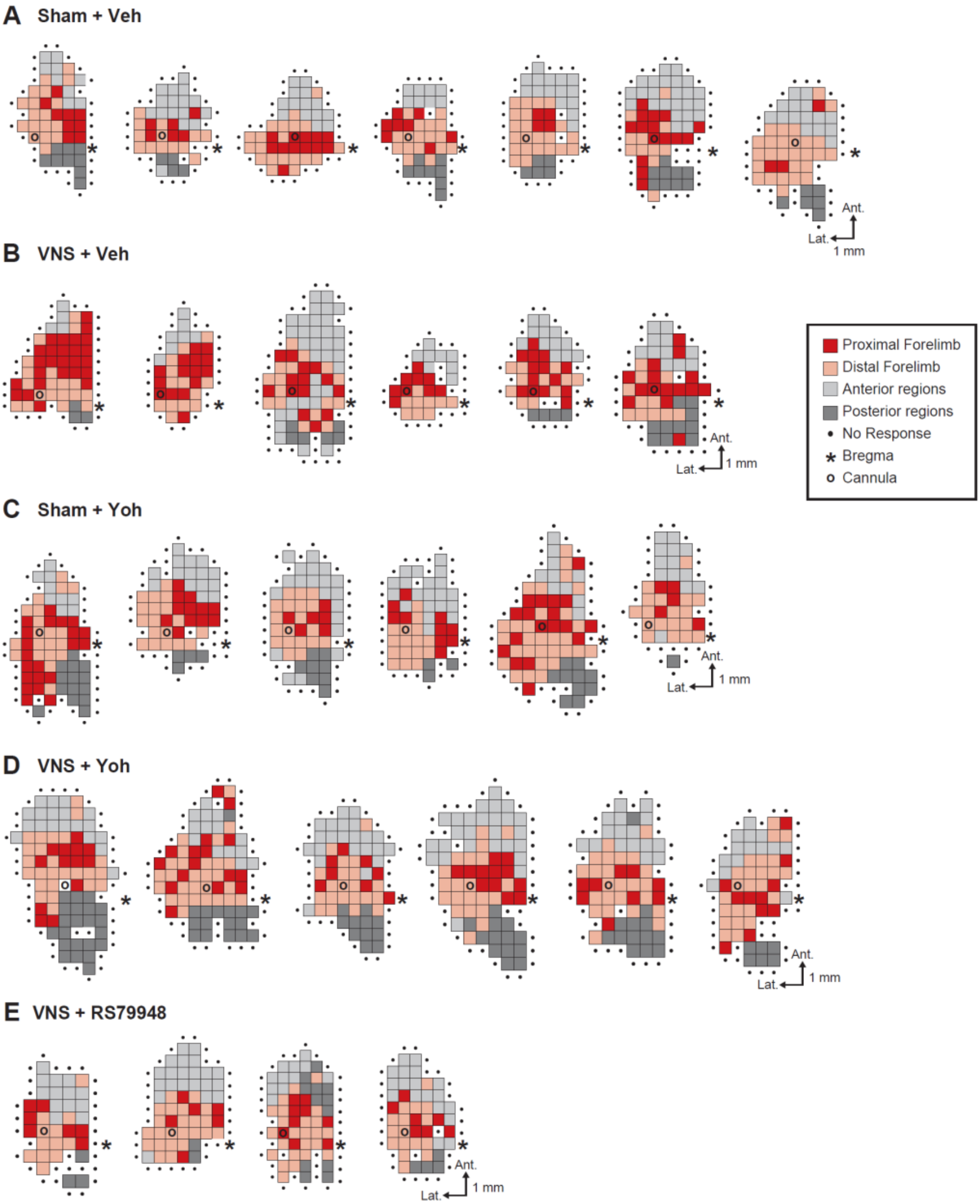

Supplementary Figure 2. Somatotopic cortical motor maps for all subjects.

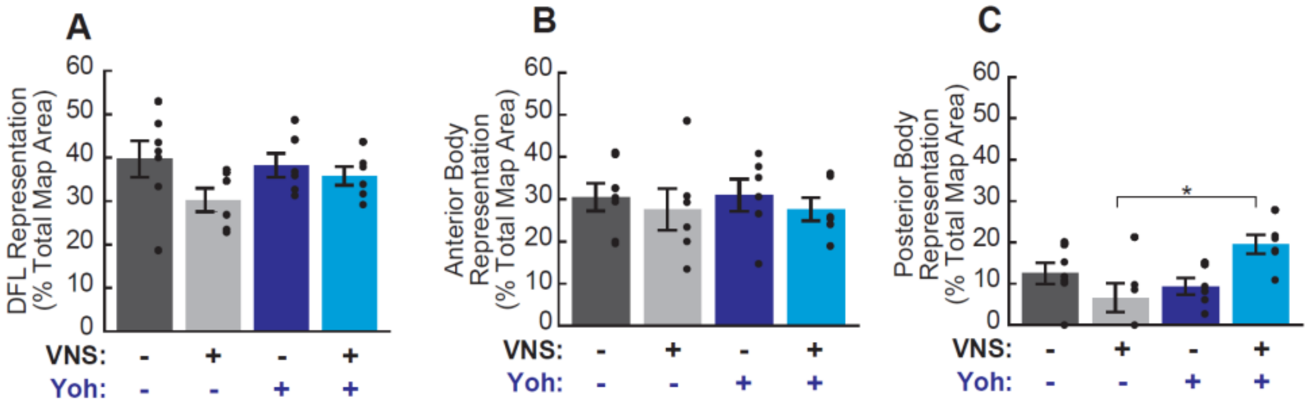

Supplementary Figure 3. Intracortical infusion of the α2-AR antagonist yohimbine did not alter the motor map representations of **(A)** distal forelimb (DFL) or **(B)** anterior body (vibrissa + jaw + neck). **(C)** The significant enlargement in total motor map size observed in the VNS|yoh group (see main Fig. 2C) was attributable to a significant enlargement of the posterior body representation (trunk + hindlimb + tail) in M1.

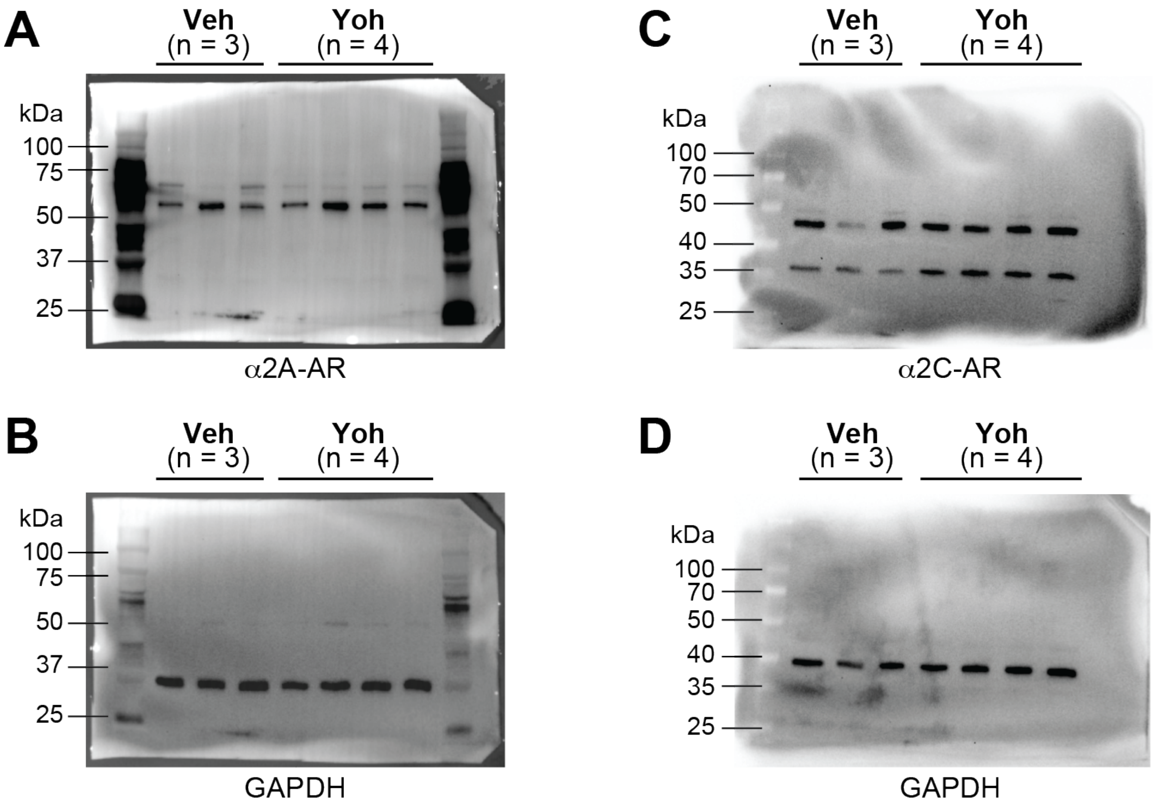

Supplementary Figure 4. Full blot images used to quantify α2-ARs expression in naïve rats following intracortical vehicle (Veh) or yohimbine (Yoh) infusions. α2A-AR and α2C-AR were processed in parallel on separate gels and expression was analyzed separately relative to same-gel GAPDH expression. **(A-B)** α2A-AR (A; 49-51 kDa) and corresponding GAPDH (B; 36 kDa) signals. **(C-D)** α2C-AR (C; 49-51 kDa) and corresponding GAPDH (D; 36 kDa) signals. Images of the blots and the chemiluminescent images were merged using Bio-Rad Image Lab software.

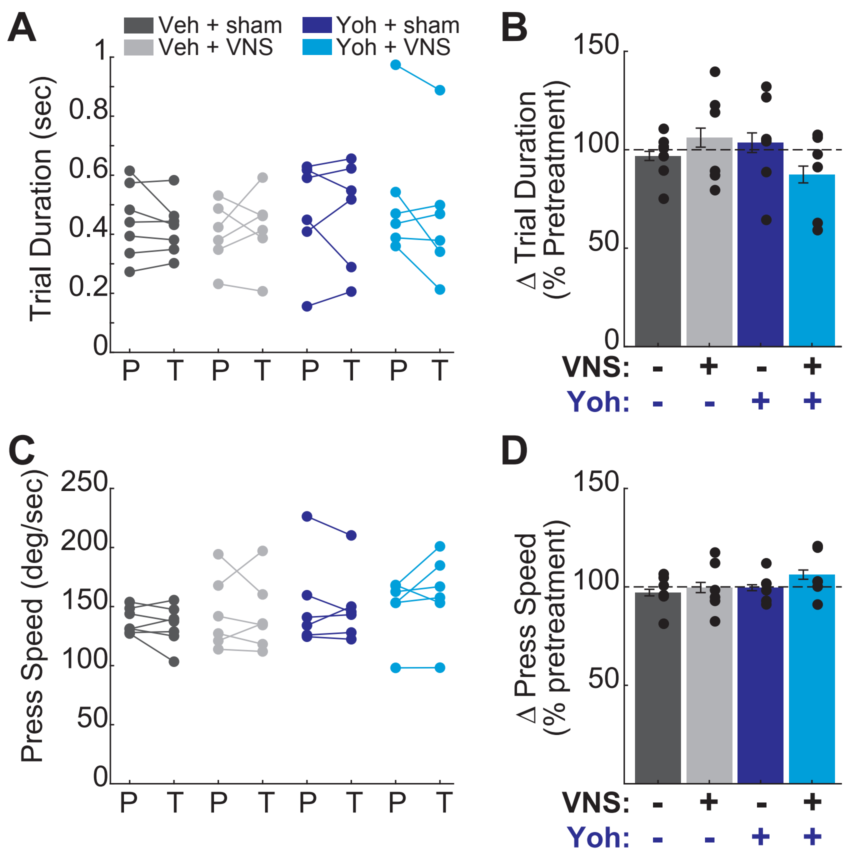

Supplementary Figure 5. Treatment did not alter coordinated motor performance. **(A)** Average trial durations did not differ between treatment groups during either pretreatment (P) or treatment (T) training epochs. **(B)** No group exhibited a significant change in trial duration during treatment. **(C)** Average lever pressing speed did not differ between treatment groups during either the pretreatment or treatment training epochs. **(D)** No group exhibited a significant change in lever pressing speed during treatment. In B & D, behavioral performance during treatment is plotted as a percentage of pre-treatment performance. Filled circles show data for individual subjects.

Table S1: Results of Tukey post-hoc comparisons of total and subregion-specific motor map areas between groups. Significant between-group differences are denoted in **bold red** for $p < 0.05$. Non-significant trends are denoted in *red italics* for $p < 0.1$. Total motor map area was significantly larger in rats that received both VNS and yohimbine infusions, due to an increase in distal forelimb (DFL) and posterior body (trunk, hindlimb, and tail) representations.

Total Motor Map Area			
	**VNS|veh**	**Sham|yoh**	**VNS|yoh**
**Sham|veh**	0.994	0.695	**0.013**
**VNS|veh**		0.570	**0.010**
**Sham|yoh**			0.146
PFL Area			
	**VNS|veh**	**Sham|yoh**	**VNS|yoh**
**Sham|veh**	*0.064*	0.514	0.623
**VNS|veh**		0.620	0.513
**Sham|yoh**			0.998
DFL Area			
	**VNS|veh**	**Sham|yoh**	**VNS|yoh**
**Sham|veh**	0.186	0.867	0.136
**VNS|veh**		*0.052*	**0.002**
**Sham|yoh**			0.485
Anterior Body Area			
	**VNS|veh**	**Sham|yoh**	**VNS|yoh**
**Sham|veh**	0.966	1.00	0.711
**VNS|veh**		0.966	0.468
**Sham|yoh**			0.744
Posterior Body Area			
	**VNS|veh**	**Sham|yoh**	**VNS|yoh**
**Sham|veh**	0.836	0.987	**0.023**
**VNS|veh**		0.676	**0.005**
**Sham|yoh**			*0.057*

Table S2: Results of Tukey post-hoc comparisons of normalized subregion-specific motor map representations. Significant between-group differences are denoted in **bold red** for $p < 0.05$. Intracortical infusions of yohimbine blocked VNS-driven expansion of the task-relevant proximal forelimb (PFL) representation in M1.

PFL Representation (% Total Map)			
	**VNS|veh**	**Sham|yoh**	**VNS|yoh**
**Sham|veh**	**0.002**	0.764	1.00
**VNS|veh**		**0.024**	**0.002**
**Sham|yoh**			0.753
DFL Representation (% Total Map)			
	**VNS|veh**	**Sham|yoh**	**VNS|yoh**
**Sham|veh**	0.176	0.987	0.820
**VNS|veh**		0.332	0.625
**Sham|yoh**			0.953
Anterior Body Representation (% Total Map)			
	**VNS|veh**	**Sham|yoh**	**VNS|yoh**
**Sham|veh**	0.906	0.991	0.959
**VNS|veh**		0.982	0.998
**Sham|yoh**			0.997
Posterior Body Representation (% Total Map)			
	**VNS|veh**	**Sham|yoh**	**VNS|yoh**
**Sham|veh**	0.575	0.992	0.288
**VNS|veh**		0.763	**0.031**
**Sham|yoh**			0.207

Table S3: Results of Tukey post-hoc comparisons of raw and normalized motor map areas between RS79948 and other treatment groups. Unlike rats treated with yohimbine + VNS, infusion of the more selective α2 antagonist RS79948 did not result in an expansion of the total map area. Cortical infusion of RS79948 did, however, block the expansion of the task-relevant proximal forelimb (PFL) representation of the motor map. DFL: distal forelimb. Significant differences are denoted in **red bold** for $p < 0.05$. Non-significant trend is denoted in *red italics* for $p < 0.1$.

	VNS|RS79948 versus…			
	**sham|veh**	**VNS|veh**	**sham|yoh**	**VNS|yoh**
Total Motor Map Area (mm²)	1.000	0.9988	0.8857	**0.0486**
PFL Area (mm²)	0.9980	*0.0764*	0.5237	0.6252
DFL Area (mm²)	0.9998	0.3493	0.9885	0.4408
Anterior Area (mm²)	0.9304	0.4405	0.6608	0.7865
Posterior Area (mm²)	1.000	0.9892	0.9985	0.9125
PFL Representation (% Total Map)	0.9975	**0.0029**	0.7510	0.9989
DFL Representation (% Total Map)	1.000	0.3855	0.9996	0.9563
Anterior Body Representation (% Total Map)	0.9158	0.5832	0.5077	0.9867
Posterior Body Representation (% Total Map)	1.000	0.9923	1.000	0.9841

Table S4: ICMS threshold current amplitudes were not altered by VNS or α2-AR antagonism.

	Vehicle		Yohimbine		2-way ANOVA		
	Sham	VNS	Sham	VNS			
	Group Mean (SEM)				p_{Drug} [F_{Drug}]	p_{VNS} [F_{VNS}]	p_{VNS} [F_{VNS}]
Total Map (μA)	107.4 (6.9)	122.4 (12.1)	122.2 (6.4)	120.8 (8.7)	0.458 [0.57]	0.444 [0.61]	0.357 [0.89]
Proximal Forelimb (μA)	89.6 (9.5)	109.6 (10.1)	106.4 (7.9)	105.5 (15.8)	0.574 [0.33]	0.401 [0.73]	0.359 [0.88]
Distal Forelimb (μA)	109.6 (6.6)	127.9 (10.0)	127.1 (9.1)	118.8 (12.0)	0.661 [0.20]	0.602 0.28]	0.174 [1.99]
Anterior Body (μA)	107.5 (8.4)	130.1 (19.2)	116.2 (7.5)	122.1 (8.6)	0.975 [0.00]	0.237 [1.48]	0.484 [0.51]
Posterior Body (μA)	141.6 (10.2)	156.4 (13.4)	157.0 (14.2)	130.1 (7.6)	0.664 [0.19]	0.628 [0.24]	0.108 [2.85]
